# Increasing the productivity and quality of cucumber crop by improving the performance of the evaporative cooling system

**DOI:** 10.1016/j.heliyon.2024.e36997

**Published:** 2024-08-29

**Authors:** Mohamed A. Rashwan, Ibrahim M. Al-Helal, Sulaiman S. Al-Showaiman, Adil A. Fickak, Waleed A. Almasoud, Fahad N. Alkoaik, Mansour N. Ibrahim

**Affiliations:** a Department of Agricultural Engineering, College of Food and Agriculture Sciences, King Saud University, P.O. Box 2460, Riyadh, 11451, Kingdom of Saudi Arabia; b Department of Agricultural & Biosystems Engineering, Faculty of Agriculture, Alexandria University, Alexandria, 21545, Egypt

**Keywords:** Developed evaporative cooling system, Plant thermal stress, Plant wilting, Dry and fresh weight, Vegetative growth, Cucumber quality

## Abstract

Evaporative cooling in the kingdom of Saudi Arabia is one of the most important cooling systems used inside greenhouses to obtain an acceptable temperature change in hot, dry climates. It is considered insufficient during very hot summer periods, when temperatures outside the greenhouse reach approximately 48 °C, which affects the efficiency of cooling, and thus, creates stress on the plants, leading to wilting and a decrease in the production rate. The cooling system is developed by making an insulated rectangular tunnel. The air comes out through vertical openings directly to the plants. The results of evaluating plant heat stress in the developed cooling system (DCS) showed an increase in the values of the photosynthesis rate, transpiration rate, carbon dioxide exchange, and stomatal conductance. The plants also appeared well in terms of the shape of the leaves, their freshness, the abundance of flowers, and the large size of the fruits, while in the traditional cooling system (TCS) the plants exhibited some wilting and some brown spots. The hectare yield reached 42.49 ton/ha for the DCS system, while it reached 37.53 ton/ha for the TCS with an increasing rate of 13.22 %. The total weight of fruits harvested within 60 days of cultivation was 4.25 kg/m^2^ for the DCS and 3.75 kg/m^2^ for the TCS. The dry and fresh weight of fruit, stem, and leaves, total dissolved solids, vitamin C, chlorophyll percentage in leaves, and total plant acidity, were higher in DCS compared to the TCS.

**Table:** Nomenclature

PVC	Poly Vinyl Chloride
DCS	Developed cooling system
TCS	Traditional cooling system
ECS	Evaporative cooling system
DECS	Direct evaporative cooling system
IECS	Indirect Evaporative cooling systems
*T*_d_	Dry Bulb Temperature, °C
*T*_w_	Wet Bulb Temperature, °C
*RH*	Relative humidity, %
*T*a	Ambient Dry Bulb temperature, °C
*V*	Air velocity, m/s
*ṁ*	Air rate, m^3^/s
*T*_do_	Dry temperature outside the greenhouse, °C
*T*_di_	Dry temperature inside the greenhouse, °C
*T*_wo_	Wet temperature outside the greenhouse, °C
η	Wet bulb effictiveness, %
*T*_*i*_	dry temperature of the air before entering the evaporative cooler, °C
*T*_*o*_	dry temperature of the air leaving the evaporative cooler, °C
*T*_*w*_	The moist temperature of the air before it enters the evaporative cooler, °C.
*TA*	Total plant titrated acidity, %
TDS	Total dissolved solids, %

## 1 Introduction

Solar radiation is a free and very beneficial input to most sectors such as heat, health, agriculture and energy production, and plays an important role in the sustainability of biological resources and chemical processes in nature [1]. In this context, knowledge of solar radiation data or estimating it as accurately as possible is vital to get the most out of the sun [1], and when designing the cooling systems [2]. On the other hand, the increased concentration of CO_2_ and other greenhouse gasses in atmosphere is causing a future climate with higher temperatures [3], and extremely high greenhouse temperature stress, or heat stress are a major problem for plant growth and productivity, especially in arid and semi-arid regions with high solar radiation values [4,5]. The high temperature is one of the non-vital factors that have a great impact on the productivity of various agricultural crops in hot regions, which affects the national economy in many countries [6,7]. It highlights the need to develope adaptation strategies and agricultural policies capable of mitigating the effects of heat stress on global food supplies [8]. In order to deal with and remedy this situation, there are different types of technologies used to cool greenhouses [9]. The most important of these technologies are active cooling methods such as direct evaporative cooling system (DECS), indirect evaporative cooling system (IECS), and two-stage evaporative cooling [[10], [11], [12]]. The DECS is an adiabatic refrigeration process in which sensible heat is converted into latent heat, the enthalpy remains constant, the specific and relative humidity increases, and the dry bulb temperature decreases. Through the DECS, heated outside air passes over a moistened porous pad usually made of chemically treated wood chips [13,14]. The amount of evaporated water is directly proportional to the surface area of wood chips [14,15]. Evaporation also depends on the amount of air passing over the wetted surface through the wood chips, as the amount of evaporated water increases with the increase in air velocity [14,15]. The combination of evaporative cooling system (ECS) with natural and forced ventilation provides a suitable climate to improve crop productivity [16]. The final temperature of the evaporative cooling process is the main goal, which is practically difficult to reach easily. Therefore, ECS must be developed to reach the optimum temperature with less energy consumption [17]. Many attempts have been made in recent years to study the performance of air coolers that operate using the DECS method in greenhouses. They tried to develop them by improving their performance to maintain high levels of air humidity, which reduces water and thermal stress on plants. Thus, reducing the possibilities of wilting and thermal exposure to plants and animals [18]. Willits [19], reported that crop production is negatively affected even when the daytime greenhouse air temperature is 1–2 °C above the optimum crop temperature. Al-Helal and Abdel-Ghany [20], found that, under extreme arid summer conditions in the central region of Saudi Arabia, (the outside radiation flux was about 1100 W m^−2^ at noon), a single stage evaporative cooler having a new pad could reduce the greenhouse air temperature by about 12 °C and increased the relative humidity by about 30 %. However, with high temperature and humidity levels, the wet-bulb temperature increases which reduces the evaporative cooling efficiency [21]. A relative humidity inside the greenhouse ranging between 80 and 90 % during the day, and between 65 and 75 % during the night, is generally recommended, but is difficult to reach technically [22]. Zhan et al. [23], has proven that with increasing temperatures, humidity increases, which leads to stress for plants or animals inside the greenhouse, which directly affects productivity and vital processes. Rosa et al. [24], made a test to prove the effect of heat stress on the growth of tomato plants inside the greenhouse, they tested two different types of plants at temperatures (25 °C–35 °C), and the results indicated that the plant leaves wilted in an attempt to resist stress at a temperature of 35 °C. While, the results showed the ideal shape and quality of the plant and fruit at a temperature of 25 °C. Generally, it is preferable to use ECS, which are economical, pollution-free, and easy to maintain, and reduce energy consumption in buildings such as greenhouses and broiler chicken houses [13,25]. Although, ECS is an effective method for reducing the rise in temperatures in the summer inside greenhouses however, in the case of very high temperatures (above 50 °C), the ECS becomes ineffective, and some plants are exposed to heat stress, causing them to wilt and die, leading to the loss of a large portion of the crop [26]. Therefore, the aim of this research is to develop the ECS inside greenhouses. So that it contributes more effectively in distributing cold air saturated with water directly to the plant sites, especially in light of the hot and dry conditions experienced by the Riyadh region, especially during the months of July and August of the summer season, in order to increase crop productivity and increasing its quality.

## 2 Materials and methods

### 2.1 The greenhouse setup for planting

**Fig. 1:**
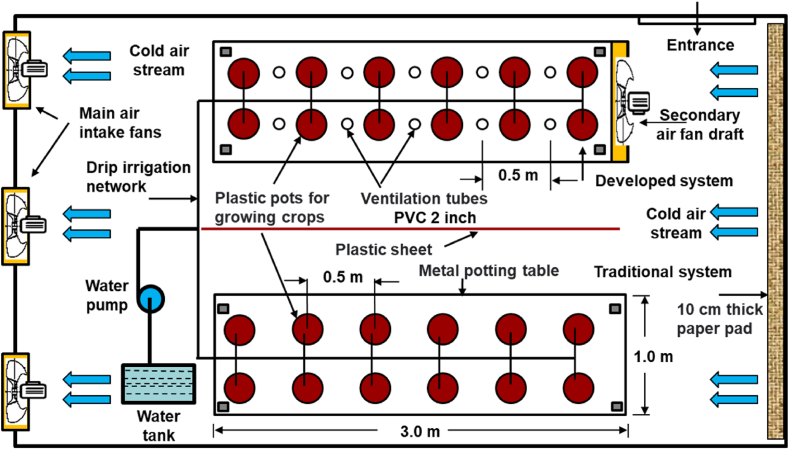
A horizontal schematic diagram showing the placement of the tables, planting pots and the drip irrigation network.

The research was conducted in one of the greenhouses located in the educational farm at the College of Food and Agricultural Sciences at King Saud University in Riyadh. The greenhouse is located in an east-west direction with a metal structure in the form of a symmetrical truss on both sides of the roof. The greenhouse has 8.3 m in length, 4.3 m in width, and the height of the roof top is 3.6 m, and covered with a layer of transparent white low-density polyethylene with a thickness of (200 μm). The greenhouse is equipped with an ECS with cellulose paper pads (4.3 m wide, 1 m hight and 0.1 m thick.) and three ventilation fans on the opposite side with a ventilation rate of 46.7 m^3^/min per fan. Outside the greenhouse, there is a 1 m^3^ water tank equipped with a 0.5 horsepower submersible pump. The submersible pump pushes water through Poly Vinyl Chloride (PVC) pipes (0.5 inche diameter), to distribute the water on the cooling pads on a regular basis. The fans and pump operate under temperature control using a thermostat equipped with a temperature sensor and a circuit breaker, which are set to obtain a temperature between 21 °C and 27 °C inside the greenhouse. Two Table (3 × 1 m^2^ each) were placed in parallel inside the greenhouse separated by a polyethylene wall, (Fig. 1), the distance between tables was 1.80 m. One of them represented the proposed developed system, as it was closed tightly on all sides, with a fan placed in the front to pull the cold air and push it into the ventilation tubes, and the other table was left as it is. An equal number of planting pots were placed on each table. A network of pipes was connected to the pots for the purpose of drip irrigation. Figure (1) represents A schematic diagram of the greenhouse, showing the placement of the tables, planting pots and the drip irrigation network.

**Table (1):** Measuring of dry and wet temperatures inside and outside the greenhouse during the day (September 2021), and calculation of the ECS efficiency in the greenhouse.

Parameter	Average air temperatures, °C	
	TCS	DCS
Dry temperature outside the greenhouse, Tdo (°C)	27.4 ± 0.1	27.4 ± 0.1
Dry temperature inside the greenhouse, Tdi (°C)	22.1 ± 0.1	21.7 ± 0.17
Wet temperature outside the greenhouse, Two (°C)	21 ± 0.1	21 ± 0.1
Wet bulb effictiveness, η (%)	82.8	89
Average RH outside the green house	26.41–77.63 % Day - Night

**Table (2):** Evaluation of plant heat stress in the TCS and DCS after 60 days of cultivation.

Measurement element	TCS	DCS
Photosynthetic rate (micromole CO_2_/m^2^.s)	16.6 ± 0.29	0.18 ± 18.5
Transpiration rate (mmol. H_2_O/mol)	2.8 ± 0.08	0.13 ± 3.35
Exchanged CO_2_ (μmol CO_2_/mol)	210.25 ± 2.22	2.99 ± 225.75
Stomatal conduction (mmol CO_2_/m^2^.s)	1.46 ± 0.02	0.05 ± 1.71
Visual examination (eye examination)	Some wilting	good

**Table (3):** Productivity measurements of cucumber plants in the TCS and DCS after 60 days of seedling.

Measurement element	TCS	DCS
Number of fruits	147	171
Total production (kg/m^2^)	3.753	4.249
Fresh weight of fruit (g)	116 ± 1.83	117.5 ± 2.38
Dry weight of fruits (g)	6.63 ± 0.13	7.05 ± 0.13
Dry plant biomass (g)	48.85 ± 0.39	51.93 ± 0.26
Fresh plant biomass (g)	535.5 ± 2.65	559 ± 2.94
Stem dry weight (g)	12.5 ± 0.18	13.5 ± 0.29
Fresh weight of stalk (g)	109.5 ± 2.38	116 ± 2.16
Dry weight of leaves (g)	36.35 ± 0.26	38.43 ± 0.17
Fresh weight of leaves (g)	426 ± 2.16	443 ± 4.97

### 2.2 Installing the proposed (developed) evaporative cooling system

**Fig. 2:**
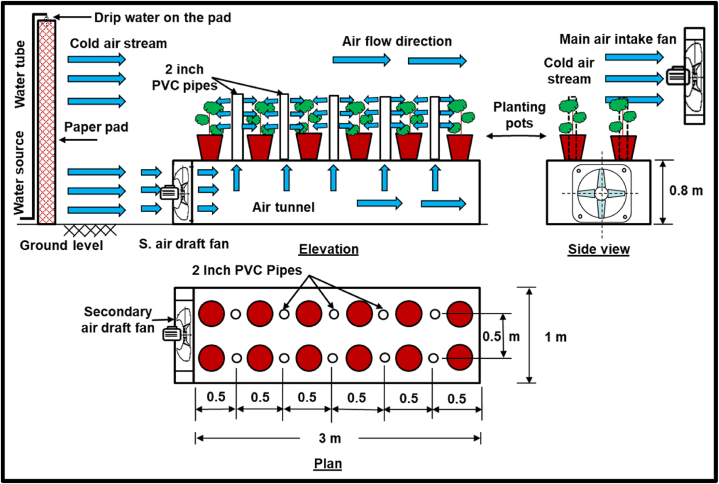
Schematic diagram showing the developed evaporative cooling system.

The developed cooling system (DCS) (Fig. 2) is based on the construction of a tunnel with a rectangular section (1 m-wide and 0.8 m-hight) through which the cool air coming from the pad passes through an intake fan placed at the front of the tunnel. Plants are grown in pots that are placed on top of the roof of the tunnel, and the air exits through openings in the roof of the tunnel, on which short vertical tubes are installed to distribute the air among the plants around it. The height of the tubes can be increased according to the height of the plants. The DCS consists of a tunnel with a rectangular section, is made of iron and the sides of the tunnel were completed using wood (0.01 m-thick.). The tunnel was insulated from the inside with polyethylene covers to prevent air leakage, and a fan was installed on the opening front tunnel facing the pad, where it draws air from in front of the main pad of the greenhouse and pushes it into the tunnel, while the other side of the tunnel is completely closed to force the air out through the vertical pipes (2 inches-diameter).

**Fig. 3:**
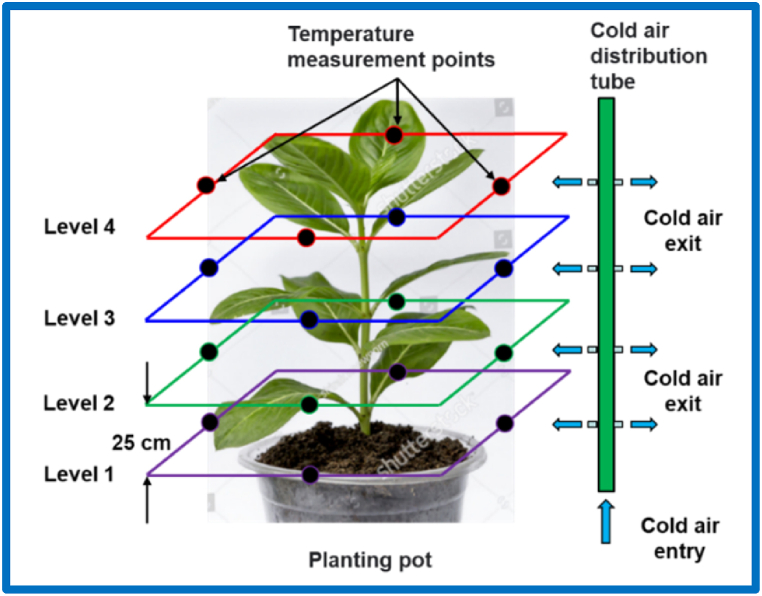
Temperature measurement points across the four levels.

Two tables were prepared for this purpose inside the greenhouse, one was provided with the proposed system and the other with the traditional cooling system (TCS), where plant pots are placed on top of them. A comparison was made between average temperatures, humidity levels and average crop yields for each of the two systems through several horizontal and vertical levels around the plants (Fig. 3). The cultivation process was carried out using two-week-old seedlings with two to three true leaves, which were brought from the nursery of the college farm in Dirab. Seedlings were planted inside the greenhouse in September of 2021. The seedlings were irrigated using drip irrigation, as a main irrigation line was installed for each table, and secondary lines for the pots branched from it every 50 cm. This technology allows each plant to receive the same amount of water.

### 2.3 Cucumber plant cultivation in the greenhouse

Cucumber is one of the plants characterized by its rapid growth, and its harvested more than once throughout the year. It is grown in the seed way and need a moderate atmosphere. In this study, the cucumber crop (Cucumber AKINCI F1 cultivar from the Turkish company Argeto) was grown in pots (30 cm diameter) placed above the tables. Various agricultural operations were carried out according to the growth stage of the cucumber plants, including pruning, where the lateral buds and flowers of the lower five true leaves of the plants were removed in order to encourage vegetative growth and ensure that the fruits do not come into contact with the soil. The plants were also hung with the crop holder, where the string was tied with a wide tie from the bottom of the stem, and its other end was tied to a wire at a height of 2 m above the table. When the plant began to grow, it was sprayed with pesticides and fertilizers were added at a rate of three days each week, whether dry fertilizers or a solution dissolved with water at a specific concentration manually. As for the irrigation procedures, they were completed automatically, as the water was opened for 5 min in the morning and evening. All agricultural operations were carried out in the greenhouse, from soil preparation, plant pruning, irrigation and fertilization until the end of the experiment, according to Ref. [27].

### 2.4 The devices and measurements

#### 2.4.1 Temperature and relative humidity measurements inside and outside the greenhouse and around the plants

Dry (T_d_) and wet temperatures (T_w_) and relative humidity (RH) were measured inside and outside the greenhouse and at the air exit openings (every hour from 8 Am to 7 *Pm*) using thermocouple wires (Type T) connected with portable devices combined with data logger (OM-EL-ESB-2-LSD, Omega Inc, USA). In addition, temperatures were measured around a random number of pots (4 pots) from the four sides surrounding the plant through 4 vertical levels as shown in Figure (3). Temperature measurement begins at the beginning of the plant's emergence, and as the height of the plant increases, points are moved to the upper level. The distance between the vertical levels is 25 cm, and the measuring points are 5 cm away from the plants in the horizontal plane.

#### 2.4.2 The rate of air entering and leaving the tunnel

The air velocity, *V* (m/s), and air rate, *ṁ* (m^3^/sec) before entering the tunnel as well as the air flow rate from the air outlet pipes were measured by the anemometer pen (850021 Pen Style Meter RH Anemometer Pen).

#### 2.4.3 Cooling efficiency of the traditional and developed cooling system

The cooling efficiency of the TCS and DCS cooling systems inside the greenhouse was calculated by applying Equation No. (1) based on dry and moist air temperatures before and after the pad [28].

$$\eta =\frac{Ti-To\;}{Ti-Tw}\times 100$$

where: *η*: wet bulb effictiveness (%), *Ti*: dry temperature of the air before entering the evaporative cooler (°C), *To*: dry temperature of the air leaving the evaporative cooler (°C), *Tw*: The moist temperature of the air before it enters the evaporative cooler (°C).

#### 2.4.4 Plant heat stress evaluation

The high temperature leads to an increase in the process of catabolism in the plant, which means an increase in the process of respiration, and also, leads to increase the plant's loss of water through the process of evaporation or the process of transpiration, in which the amount of water lost is greater than the amount of water absorbed, which leads to dehydration, wilting and death of the plant. Therefore, the heat stress of the plant was evaluated by measuring a set of elements and characteristics like: photosynthesis, rate of respiration, transpiration, exchanged carbon dioxide, and stomatal conductance using the portable photosynthesis device LI-6400Xt.

#### 2.4.5 Visual examination of plant growth and crop characteristics

An outward examination of the growing plants in pots was carried out in terms of leaf shape, leaf area, number, plant height, number of fruits, and number of flowers, to make a comparison between the two systems.

#### 2.4.6 Vegetative growth, quality characteristics and crop yield

Four plants were randomly selected from each table to conduct phenotypic measurements and the vegetative and fruiting growth of cucumber plants in the greenhouse as follows.

##### 2.4.6.1 External characteristics and vegetative growth

Different characteristics of plants were measured, such as: Plant height (cm), Paper area (cm^2^, by measuring device, LI-COR 3000C), Stem diameter (mm, by a digital vernier caliper), Plant fresh weight (g), Dry weight of leaves and stem (gm, by measuring plant weight after drying a fresh sample of leaves and stem in a drying oven at 70 °C for 48–72 h until the weight becomes constant).

##### 2.4.6.2 Productivity of cucumber plants

All measurements of yield were taken in the vegetable laboratory, Department of Plant Production, College of Food and Agricultural Sciences, and the measurement included:

**Number of fruits:** the total number of all harvested fruits during the crop cycle was recorded in each table, and then the average number of fruits for each plant was calculated.

**Total production (kg/m**^**2**^**):** The crop was weighed (kg) for all growing plants on each table after the harvest process. It was considered that, the fruits were full of the size characteristic of the variety, and the harvest was done manually by picking the fruits from the branches with sharp scissors, leaving a small part of the branch attached to the fruit [29]. The weights were recorded for each table separately every 3 days during the experimental period.

**Fruit fresh weight (g):** The average fruit fresh weight in grams was calculated by dividing the total weight of harvested fruits by the total number.

**Dry weight of the fruit (gm):** The average dry weight of the fruit was calculated by dividing the total weight of the harvested fruits (after drying them in an oven at 70 °C until the weight becomes stable through 48–72 h) by the total number.

##### 2.4.6.3 Quality characteristics of cucumber fruits

All measurements of the quality of cucumber fruits were measured in the vegetable laboratory, Department of Plant Production, College of Food and Agricultural Sciences. The measurements included.

## 3 Natural characteristics

Fruit length, fruit diameter, fruit color, and fruit weight were measured using rular and scale.

## 4 Chemical properties

**Vitamin C (mg/100g fresh weight):** The ascorbic (vitamin C) content of cucumber fruit juice was measured by the titrimetric method [30].

**Total plant titrated acidity (TA, %):** The acidity of cucumber fruits was measured by titration according to A.O.A.C, [30].

**Total dissolved solids (TDS, %):** The percentage of TDS in cucumber juice was calculated using a device (Digital portable refractometer.101 model, ATAGO Japan) by placing a drop of fruit juice on a lens in the device and recording the reading shown on the screen of the device.

## 5 Results and discussion

### 5.1 Temperatures and relative humidity measurement inside and outside the greenhouse

**Fig. 4:**
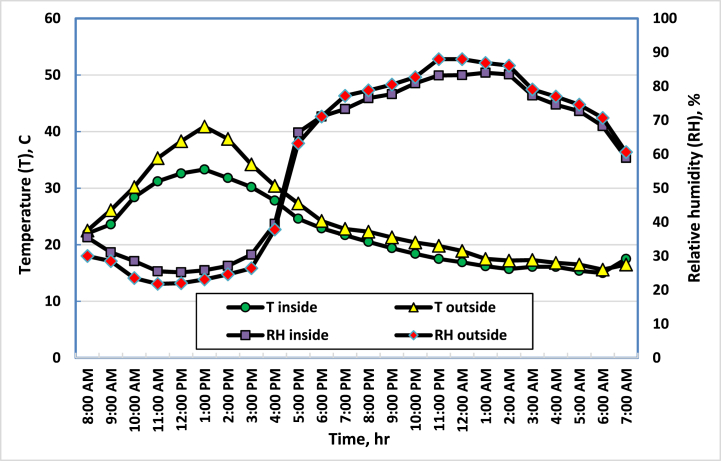
Average temperatures and RH during mid-September in the center and outside of the greenhouse.

The temperature and relative humidity (RH) of the air are interdependent parameters that should be controlled simultaneously. These two parameters strongly affect the growth of crops [31]. The temperatures and relative humidity of the air inside and outside the greenhouse were measured, recorded on an hourly average, and kept in the data logger. The measurement process took place over a period of 23 days after transplanting during the months of September and October of 2021. The results showed that there are differences in temperature and RH inside the greenhouse (T1, RH1) and outside the greenhouse (T2, RH2), depending on the change in daily temperatures and RH. In general, the air temperatures during the day were always higher than the temperatures in the morning and evening. It has been observed that there is an inverse relationship between the air temperature and its RH. Figure (4) represents the temperature and RH of the air in the middle of the greenhouse during the operation of the evaporative cooling system (ECS), compared to the air temperature and RH outside the greenhouse. It is clear that, the temperature inside the greenhouse is relatively high, there is a temperature difference that sometimes reaches 5 °C during the daytime period between the outside and the inside, and there is a difference in the RH of the air inside and outside the greenhouse that reaches more than 7 % during the daytime period as a result of the operation of the ECS. On the other hand, cucumbers thrive best at relatively high temperatures between 25 and 30 °C, and they must be well supplied with moisture and nutrients throughout the growing season [32].

### 5.2 Temperatures and relative humidity measurement around plants

**Fig. 5:**
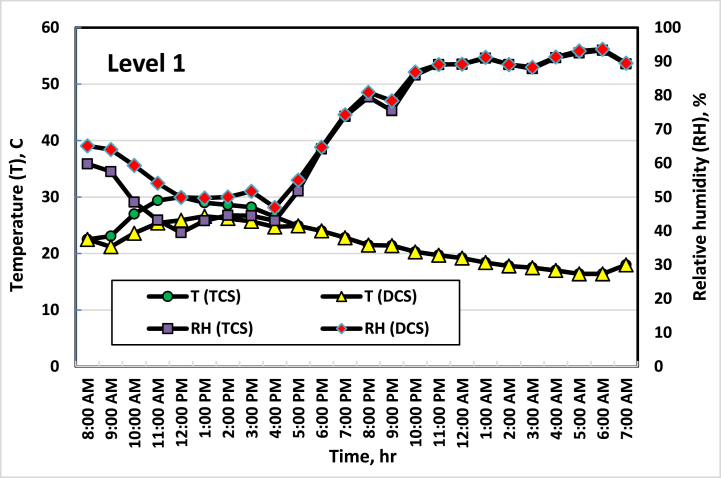
Average temperatures and RH at the first level (beginning of the pot). Sep. 7, 2021.

**Fig. 6:**
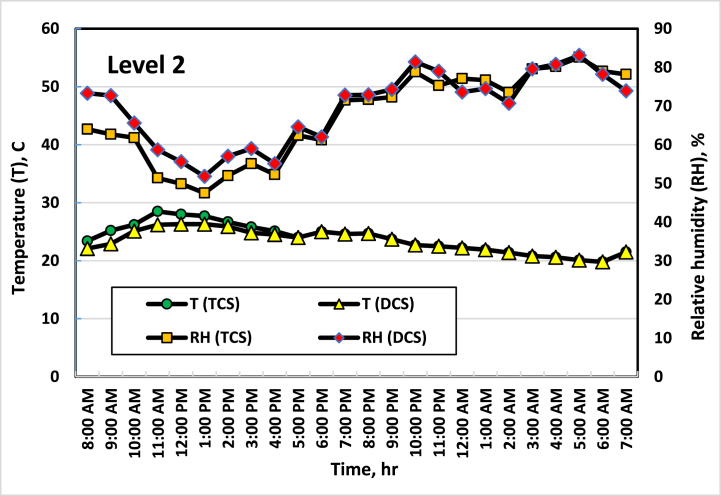
Average temperatures and RH at the second level (25 cm height from the beginning of the pot). Sep. 21, 2021.

**Fig. 7:**
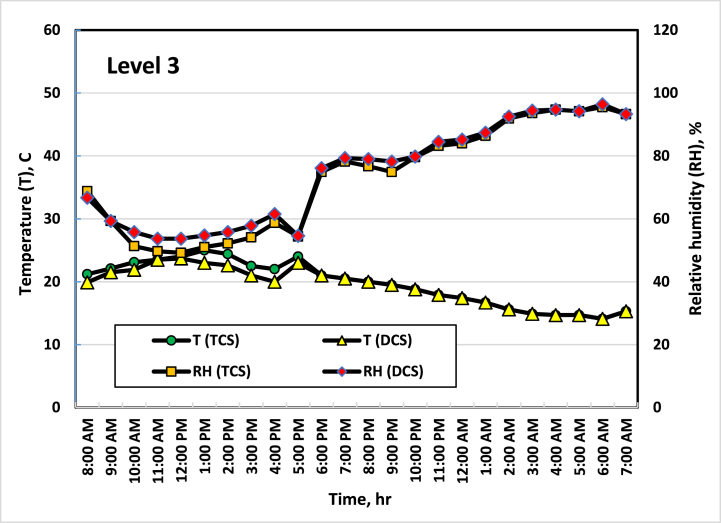
Average temperatures and RH at the third level (50 cm height from the beginning of the pot). Oct. 6, 2021.

**Fig. 8:**
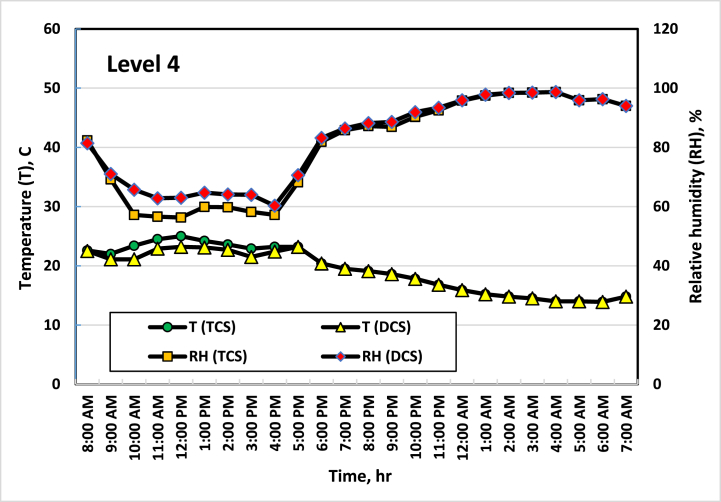
Average temperatures and RH at the fourth level (75 cm height from the beginning of the pot). Oct. 20, 2021.

Fig. 5, Fig. 6, Fig. 7, Fig. 8 show the average temperatures and RH across the four vertical levels of measurement for both the TCS and DCS. The first measurement level starts at the upper edge of the pot, then the next measurement levels are 25 cm away from the previous level. The temperature and RH sensors are raised to the highest level approximately every two weeks so that the measurement is consistent with plant growth. It is clear from these curves that there is a difference in temperatures between the TCS and the DCS, as the temperature of the DCS is lower (from 2 to 4 °C) than the TCS, especially during daylight hours (from 8 a.m. to 7 p.m.). This result agreed with the result obtained by Amani et al. [33], where they found that the optimal RH inside the greenhouse should range between 60 and 80 %. On the other hand, the optimum temperature for cucumber growth is between 20 and 25 °C, temperatures of around 35 °C can induce heat stress in the plants [34]. This had a good effect on the growth and flowering of plants, as the distribution and concentration of air and the increase in RH in the area surrounding the plant led to a reduction in the stress resulting from the high temperature, which led to good growth of the plants, as the leaves appeared better in the DCS than in the TCS. While in the TCS, the effect of stress appeared on some plant leaves, and some leaves appeared with yellow or brown spots, and some of them appeared in a downward curve. It is also noted that the differences in temperature in the two systems appear clearly during the day (from 8 a.m. to 4 p.m.) and then are almost equal in the evening period (from 5 p.m. to 6 a.m.). This is because the ECS stops working when the air temperature inside the greenhouse is less than 21 °C.

### 5.3 Measuring the air speed entering and leaving the tunnel

**Fig. 9:**
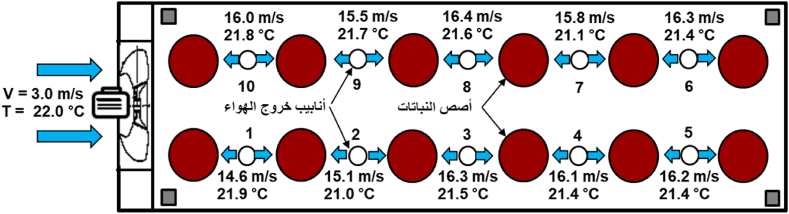
Air speed and temperature when leaving the pipes to the plant during the day (from 8 a.m. to 4 p.m.).

**Fig. 10:**
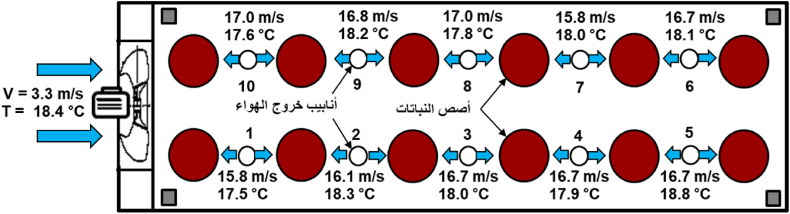
Air speed and temperature when leaving the pipes to the plants during the night (from 5 p.m. to 12 p.m.).

The presence of a current of cold air around the plants works to remove the hot air from this area, which reduces heat stress, and increases the growth [5]. The average speed of cold air that enters the DCS ranged between 3.3 m/s and 3 m/s, while there was a significant increase in the speed after exiting the vents due to the small diameter of the exit tubes. The average speed ranged between 14 m/s and 17 m/s. While the average air temperature before entering the DCS ranged between 21 and 24 °C during the day and between 15 and 20 °C during night, while the air temperature after leaving the pipes ranged between 21 and 23.6 °C during the day and between 15.5 and 20 °C during the night. Fig. 9, Fig. 10 show a horizontal diagram of the DCS within 12 h, showing the average speed and temperature of the air before entering and exit of the pipes.

### 5.4 Cooling efficiency of the developed system inside the greenhouse

For the DCS, the average temperature measured inside the tunnel was used instead of the temperature measured after the pad in the TCS. The average efficiency calculated according to the measured temperatures inside and outside the greenhouse and in the tunnel was 82.8 % and 89 % for TCS and DCS respectively as shown in Table (1). This result consistent with what was stated by Soussi et al. [5], that the cooling effect of ventilation systems is more effective when mechanical ventilation is used depending on the outside ambient temperature conditions. The DCS gave a higher efficiency due to the decrease in the temperature of the air coming out of the pipes due to the increase in its speed. This result can be explained by the fact that increasing the air speed leads to a decrease in temperature difference between the inlet and outlet, and this is because the higher speed does not give the air enough time for the heat exchange process, so the temperature of the outlet air decreases [35].

### 5.5 Plant heat stress evaluation

The results of the plant heat stress assessment as shown in Table (2), showed that all the measured elements are in favor of the DCS, as these measurements recorded some increases, albeit slight, but they are an indication of preference for the DCS. The result of the external examination was also in favor of the DCS to confirm this preference, as the plants appeared well in terms of the shape of the leaves, their freshness, the abundance of flowers, and the appearance of fruits in large sizes, while the plants appeared in the TCS with some wilting and some brown spots, which confirm the exposure of plants to stress. These results in agreement with Teixeira et al. [36], they found that, the productivity of important agricultural crops decreases significantly when exposed to short periods of high temperatures during the reproductive period.

### 5.6 Production and chemical properties of plants

**Fig. 11:**
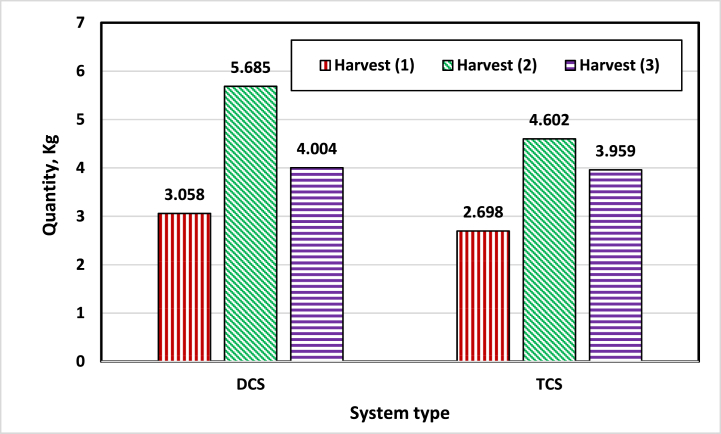
Quantity of cucumber fruit production during three harvests in both the TCS and DCS, after 60 days of cultivation.

The total weight of fruits harvested from each system within 60 days of cultivation was 11.259 kg (3.753 kg/m^2^) for the TCS and 12.747 kg (4.249 kg/m^2^) for the DCS (based on cultivation area of 3 m^2^). The hectare yield was 42.49 ton/ha for the TCS and 37.53 ton/ha for the DCS with an increase rate of 13.22 %. The results of the chemical properties of the plants for each system showed an increase in the average values in the DCS compared to the TCS. Figure (11) and Table (3) show the increase in total productivity and chemical properties of plants in the DCS compared to the TCS. This is due to the improvement of the conditions surrounding the plants to a better degree and the reduction of thermal stresses to the leaves than those in the TCS. The TCS results are consistent with Mishra et al. [7], they point out that, as a result of global warming, temperature spikes due to heat waves may become more frequent and negatively affect plant function. They added that, the effects of warming exhibited the greatest impacts on plant growth and development at different growth stages, such as the vegetative, reproductive, and grain-filling stages, ultimately affecting grain quality and overall crop quality and yield.

### 5.7 Quality traits of cucumber fruits

#### 5.7.1 Natural characteristics and vegetative growth measurement

The results of measuring the vegetative and fruit growth of the cucumber crop in the TCS and DCS after 60 days of transplanting (Table 4), showed that the measurements of plant height, fruit length, diameter, weight, and leaf area were greater in the DCS than in the TCS. Skarzy'nska et al. [37], showed that, when suitable climatic conditions are present, fruit growth depends on the interaction of genetic factors that can act as inhibitors or stimulators of growth. They lead to the diversity of fruits in terms of most basic morphological characteristics, such as shape, size and color, which are important criteria for improving fruit quality (see Table 5).

**Table (4):** The vegetative and fruiting growth of cucumber plants in the TCS and DCS after 60 days of seedling.

Measurement element	TCS	DCS
Plant height (cm)	156.75 ± 1.71	176.75 ± 1.71
Fruit length (cm)	12.8 ± 0.55	0.58 ± 13.55
Fruit diameter (cm)	2.55 ± 0.13	0.21 ± 2.85
Leg diameter (mm)	13.35 ± 0.13	0.22 ± 14.6
Fruit weight (g)	116 ± 1.83	2.38 ± 117.5
Paper area (cm^2^)	3939.9 ± 12.5	15.8 ± 4123.7

**Table (5):** Measuring the chemical characteristics of the cucumber crop for the TCS and DCS after 60 days of seedling.

Chemical characteristics	TCS	DCS
Total dissolved solids (%)	2.6 ± 0.08	0.13 ± 3.05
Vitamin C (mg/100 cm^3^ jouce)	5.65 ± 0.13	0.13 ± 6.35
Chlorophyll content in leaves (%)	43.25 ± 0.96	0.96 ± 46.75
Titratable acidity (%)	0.0125 ± 0.0003	0.0002 ± 0.0139
Transpiration (mmol. H_2_O/mol)	2.8 ± 0.08	0.13 ± 3.35
Exchanged CO_2_ (μmol CO_2_/mol)	210.25 ± 2.22	2.99 ± 225.75
Stomatal conduction (mmol CO_2_/m^2^. s)	1.46 ± 0.02	0.05 ± 1.71
Photosynthesis (micromole CO_2_/m^2^. s)	16.6 ± 0.29	0.18 ± 18.5

#### 5.7.2 Chemical properties measurement

After 60 days of seedling, four pots were taken randomly from each table, and the chemical properties were measured as shown in Table No. (5). The results showed that there are relative differences in favor of the developed system, as shown in Table No. (5). The chlorophyll content is an important indicator, showing the health status and photosynthetic capacity of the plant [6]. Also, the transpiration rate of plants is affected by the amount of moisture in the air, which inhibits plant health, growth, and development [22].

## 6 Conclusion

The developed cooling system (DCS) contributed to reducing greenhouse temperatures by up to 5 °C and increasing the RH to about 7 % compared to the air temperature and relative humidity outside. The DCS reduced temperatures and increased RH around the plant during daylight hours by 2–5 °C and from 3 % to 7 % respectively, compared to the TCS. The presence of an air current around the plant reduced heat stress processes, as the average velocity of cold air entering the DCS ranged between 3.3 m/s and 3 m/s with average temperatures between 21 and 24 °C during the daytime and between 15 and 20 °C during the night period, and the average air speed exiting the DCS ranged between 14 m/s and 17 m/s with average temperatures between 21 and 23.6 °C during the day and between 15.5 and 20 °C during the night. The cooling efficiency of the DCS reached 89 % during the day, compared to 82.8 % for the TCS, calculated based on the average temperatures measured inside and outside the greenhouse. The production of the crop was 4.249 kg/m^2^ (42.49 ton/ha) for the DCS compared with 3.753 kg/m^2^ (37.53 ton/ha) for the TCS with an increasing rate of 13.22 %. Measurements and results showed an increase in the total productivity and chemical properties of cucumber plants in the DCS compared to the TCS. In future work, water and energy consumption in the two systems will be studied to determine the extent to which the proposed system can be applied.

## Funding

This work was supported by the 10.13039/100019725Deanship of Scientific Research at 10.13039/501100002383King Saud University [Research group project No. RGP-VPP-134].

## Institutional review board statement

Not applicable.

## Informed consent statement

Not applicable.

## Data availability statement

Not available.

## CRediT authorship contribution statement

**Mohamed A. Rashwan:** Writing – review & editing, Writing – original draft, Validation. **Ibrahim M. Al-Helal:** Supervision, Investigation, Conceptualization. **Sulaiman S. Al-Showaiman:** Writing – original draft, Methodology, Investigation, Data curation. **Adil A. Fickak:** Writing – review & editing, Validation, Investigation. **Waleed A. Almasoud:** Writing – original draft, Formal analysis, Data curation. **Fahad N. Alkoaik:** Writing – review & editing. **Mansour N. Ibrahim:** Writing – review & editing.

## Declaration of competing interest

The authors declare that they have no known competing financial interests or personal relationships that could have appeared to influence the work reported in this paper.
